# 2193 The influence of health insurance stability on racial/ethnic differences in diabetes control and management

**DOI:** 10.1017/cts.2018.264

**Published:** 2018-11-21

**Authors:** Alison G. M. Brown, Nancy R. Kressin, Norma Terrin, Amresh Hanchate, Jillian Suzukida, Sucharita Kher, Lori L. Price, Amy LeClair

**Affiliations:** Tufts University

## Abstract

OBJECTIVES/SPECIFIC AIMS: The aim of this study is to examine if stable health insurance coverage is associated with improved type 2 diabetes (DM) control and with reduced racial/ethnic health disparities. METHODS/STUDY POPULATION: We utilized EMR data (2005–2013) from 2 large, urban academic health centers with a racially/ethnically diverse patient population to longitudinally examine insurance coverage, and diabetes outcomes (A1C, LDL cholesterol, BP) and management measures (e.g., A1C and BP monitoring). We categorized insurance stability status during each 6-month interval as 6 separate categories based upon type (private, public, uninsured) and continuity of insurance (continuous, switches, or gaps in coverage). We will examine the association between insurance stability status and DM outcomes adjusting for time, age, sex, comorbidities, site of care, education, and income. Additional analysis will examine if insurance stability moderates the impact of race/ethnicity on DM outcomes. RESULTS/ANTICIPATED RESULTS: Overall, we anticipate that stable health insurance coverage will improve measures for DM care, particularly for racially/ethnically diverse patients. DISCUSSION/SIGNIFICANCE OF IMPACT: The finding of an interaction between insurance stability status and race/ethnicity in improved diabetes management and control would inform the national health care policy debate on the impact of stable health insurance.

